# Bilateral Facial Palsy Without Ocular Muscle Involvement in Myasthenia Gravis: Case Report

**DOI:** 10.7759/cureus.23210

**Published:** 2022-03-16

**Authors:** Muhammad Umair, Filzah Faheem, Hashir Amin Malik, Syed Ahmad Ali Hassan, Athar Iqbal

**Affiliations:** 1 Neurology, AINeuroCare Academy, Dallas, USA; 2 Neurology, Shaikh Zayed Medical Complex, Lahore, PAK

**Keywords:** emg, nerve conduction study (ncs), facial palsy, myasthenia gravis (mg), acetylcholine receptor

## Abstract

Myasthenia gravis (MG) is an autoimmune neuromuscular junction disorder that is more common among women than men. It has two major subtypes, namely, ocular and generalized MG, and while facial weakness is common in generalized MG, facial weakness without the involvement of ocular muscle is exceedingly rare. Here, we describe the case of a middle-aged man who presented with bilateral facial palsy but without diplopia or proximal muscle weakness. The patient tested positive for acetylcholine receptor antibodies and exhibited amplitude decrement on repetitive nerve stimulation, which are diagnostic for MG. This report emphasizes the importance of neurodiagnostic and physiological testing in patients presenting with bilateral facial weakness alone.

## Introduction

Myasthenia gravis (MG) is an autoimmune disorder of the neuromuscular junction with an incidence rate of 1.7-21.3 per 100,000 person-years and a global prevalence of 5.3 per million person-years [1]. It occurs more frequently in women compared with men with a ratio of three-to-two [2]. It presents between the ages of 20-30 years in females and 50-60 years in males [3].

Two major subtypes of MG are ocular MG and generalized MG [3]. Facial weakness commonly occurs in generalized MG, but involvement of the facial nerve without ophthalmoplegia is extremely rare [4]. Here, we present a unique case of bilateral facial weakness without ophthalmoplegia in a patient with generalized MG who presented to the neurology department.

## Case presentation

A 58-year-old man presented to our outpatient department with a complaint of progressively worsening dysphagia and dysphonia for the last four weeks. His comorbidities included diabetes mellitus and hypertension, which were managed by sulfonylureas and calcium channel blockers, respectively. His dysphagia was initially limited to solids but then progressed to liquids as well. There was no reported ophthalmic involvement or limb weakness.

A neurological examination revealed bilateral facial weakness, lagophthalmos, and Bell’s phenomenon. He also exhibited difficulty in blowing his cheeks and pursing his lips, and his speech had an increasingly nasal quality. Diplopia, ptosis, eye muscle weakness, and proximal muscle weakness were not observed on serial examination. Deep tendon reflexes were intact in all limbs. Muscle strength, according to Medical Research Grading, was 5/5 in all proximal limb muscles.

Laboratory tests revealed slight hypokalemia which was corrected with two ampoules of intravenous potassium chloride. Slightly raised serum creatinine and serum lactate dehydrogenase (LDH) levels were reported (Table 1). All other laboratory parameters, such as complete blood count, liver function tests, and renal function tests were within normal limits. He tested negative for hepatitis B, hepatitis C, and HIV. Both opening pressure during lumbar puncture and subsequent CSF analysis were normal. Computed tomography (CT) of the brain was unremarkable. After obtaining written consent, a neostigmine test was performed in an ICU setting with 2.5 mg of the diluted drug, which led to a significant improvement in nasal speech and lagophthalmos. Repetitive Nerve Stimulation and Electromyography studies (Table 2) revealed an amplitude decrease in both right and left facial nerve after one, two, three and four minutes of exertion. He also tested positive for acetylcholinesterase antibodies (Table 1).

**Table 1:** Investigations mmol/L: millimole per liter; mg/dL: milligram per deciliter; U/L: units per liter; nmol/L: nanomoles per liter LDH: lactate dehydrogenase

Investigations	Result	Reference Range	Units
Serum Potassium	2.9	3.5–5.0	mmol/L
Serum Creatinine	1.7	0.6–1.3	mg/dL
Serum LDH	364	81–234	U/L
Serum Acetylcholine Receptor Antibodies	>8.00	<0.40	nmol/L

**Table 2:** Repetitive Nerve Stimulation Repetitive nerve stimulations reveal a significant decrement in the bilateral innervated facial nerves. Hence, suggesting neuromuscular junction disorder, most likely myasthenia gravis.

Right Facial Nerve					
	00 minutes after exertion	01 minutes after exertion	02 minutes after exertion	03 minutes after exertion	04 minutes after exertion
Amplitude Decrement	9.00%	10.30%	13.00%	16.00%	11.00%
Area Decrement	9.00%	10.00%	12.60%	11.70%	15.00%
Left Facial Nerve					
	00 minutes after exertion	01 minutes after exertion	02 minutes after exertion	03 minutes after exertion	04 minutes after exertion
Amplitude Decrement	17.00%	19.00%	23.00%	29.00%	24.00%
Area Decrement	10.00%	11.00%	10.70%	7.70%	13.00%

Based on the results of the clinical, laboratory, and neurophysiological evaluations, a diagnosis of MG was made, and he was prescribed pyridostigmine and prednisolone, which resulted in marked improvement of both dysphagia and dysphonia. The patient will undergo a chest CT scan with contrast to rule out the presence of a thymoma and follow-up visits will be conducted on a monthly basis. Myasthenia patients usually have a significant improvement in their muscle weakness following treatment and maintain a normal or closer to normal lifestyle [5].

## Discussion

Ophthalmoparesis typically develops within two years after the onset of MG, and about 85% of the patients suffering from MG primarily present with drooping eyelids, i.e., ptosis and weakness of eye muscles. Approximately 80% of the patients with generalized MG also suffer from weakness in muscles related to facial expression and bulbar function. In contrast, involvement of the facial muscles without weakening of the extraocular muscles is exceptionally rare [4] as drooping eyelids, ophthalmoplegia, double vision, and excessive muscle fatigue following physical activity are the main symptoms of ocular MG [2]. Uniquely, none of these ocular symptoms were present in our patient.

The acetylcholine receptor antibody test has both high sensitivity and specificity for MG, with sensitivity ranging between 80%-90% [6]. The main diagnostic criteria for MG include presence of AchR antibodies, and pharmacologic and electrodiagnostic tests. The ice-pack test, while recommended by some, is more useful for establishing ocular MG [7]. Our patient showed a positive response to neostigmine, tested positive for AchR-Ab, and displayed amplitude reduction in both facial nerves upon repeated exertions. All these findings helped to establish a definitive diagnosis of MG despite the absence of routine clinical symptoms.

The patient’s clinical condition improved markedly upon pyridostigmine and prednisolone therapy. Pyridostigmine is the preferred drug of choice in all subtypes of MG and its dosage is determined by the severity of the symptoms. Additionally, prednisone or prednisolone, coupled with azathioprine, is considered first-line immunosuppressive therapy [8].

A similar case has been described in literature wherein a 77-year-old patient had symptoms identical to those seen in our patient, i.e., dysphagia and dysphonia, as well as a bilateral facial weakness without any ocular involvement [4]. Therefore, MG should be considered as a differential diagnosis whenever patients present with bilateral facial weakness without ocular involvement. Other similar cases have been reported in literature, but involving neonatal and juvenile populations [9,10].

## Conclusions

Our case report indicates that MG can present with bulbar type and facial diplegia but without ocular and limb involvement. Hence, in patients presenting with bilateral facial nerve weakness, neurophysiological examination and electrodiagnostic tests are needed to rule out neuromuscular junction disorders, particularly generalized MG, even in the absence of ocular symptoms. Further, as very few such cases have been reported to date, we recommend mandatory screening of such patients for MG, along with neurophysiological examination and electrodiagnostic testing.
